# The Income Gap in Voting: Moderating Effects of Income Inequality and Clientelism

**DOI:** 10.1007/s11109-020-09652-z

**Published:** 2020-10-12

**Authors:** Twan Huijsmans, Arieke J. Rijken, Teodora Gaidyte

**Affiliations:** 1 Department of Sociology, University of Amsterdam, Amsterdam, The Netherlands; 2 Department of Sociology, Vrije Universiteit Amsterdam, Amsterdam, The Netherlands; 3 Faculty of Humanities, Universiteit Leiden, Leiden, The Netherlands

**Keywords:** Economic inequality, Voting, Relative income, Clientelism

## Abstract

We investigated whether income gaps in voting turnout vary with country-level economic inequality, and whether this pattern differs between wealthier and less-wealthy countries. Moreover, we investigated whether the prevalence of clientelism was the underlying mechanism that accounts for the presumed negative interaction between relative income and economic inequality at lower levels of national wealth per capita. The harmonised PolPart dataset, combining cross-national surveys from 66 countries and 292 country-years, including 510,184 individuals, was analysed using multilevel logistic regression models. We found that the positive effect of relative income on voting was weaker at higher levels of economic inequality, independent of the level of national wealth. Although clientelism partially explains why economic inequality reduces the income gap in voter turnout, it does not do so in the way we expected. It seems to decrease turnout of higher income groups, rather than increase turnout of lower income groups. Importantly, that economic inequality reduces the income gap in voter turnout does not imply that economic inequality is positive for democratic representation, since economic inequality was found to depress the likelihood of voting for all income groups.

**Electronic supplementary material:**

The online version of this article (10.1007/s11109-020-09652-z) contains supplementary material, which is available to authorized users.

## Introduction

It has been well established in the literature that “people with less vote less” (Laurison 2016, p. 684). Many studies, conducted in Western democracies, showed that the less income an individual has, the less likely he or she is to vote and participate in other forms of politics (e.g. Brady et al. 1995; Solt 2008, 2010, 2015; Jensen and Jespersen 2017; Armingeon and Schädel 2015; Hakhverdian et al. 2012; Smets and Van Ham 2013; Geys 2006). The existing research also revealed that the income gap in political participation differs *between* countries. To what extent people with different levels of income turn in ballots in a certain country depends on political and socio-economic conditions, economic inequality being among the most important ones (Solt 2008, 2010; Scervini and Segatti 2012; Jaime-Castillo 2009; Matsubayashi and Sakaiya 2018). However, the findings in the literature on how economic inequality at the country level affects the income gap in voting turnout are conflicting.

Three recent studies on the topic, that were based on a broad sample of countries, indicated that the income gap in voting is smaller in more economically unequal countries (Amat and Beramendi 2016; Jensen and Jespersen 2017; Matsubayashi and Sakaiya 2018). In those unequal countries, lower-income citizens seem to be at least as likely to vote as people from higher-income groups. This might be explained by the way how economic inequality influences mobilisation strategies of political parties (Amat and Beramendi 2016; Matsubayashi and Sakaiya 2018). Economic inequality provides an incentive for political parties to employ clientelistic mobilisation strategies. Such strategies mobilize especially the lower-income groups to vote, which decreases the income gap in voting.

On the contrary, studies that explicitly focused on advanced, relatively wealthy, democracies indicated that the income gap in voting turnout is larger in more economically unequal countries (Gallego 2015; Solt 2008, 2010). The most prominent explanation for this finding is the *relative power theory*, which assumes that more political power is in the hands of the higher income groups when economic inequality is higher. This leads people of lower income groups to perceive the political system as incapable to defend their interest, and therefore they abandon their engagement in politics.

When comparing the contrasting results of these studies, it seems like the association between economic and political inequality, and the underlying explanatory mechanisms, cannot be generalized across the world. It seems to differ between studies that only include advanced European democracies that are relatively wealthy, and studies that also include less-wealthy countries from other parts of the world. Even within Europe, the association between economic inequality and the income gap in voting does seem to depend on national wealth (Jensen and Jespersen 2017).

We aim to bring together previous studies on economic inequality and the income gap in voting by formulating separate hypotheses for wealthy and less-wealthy countries. Based on the *relative power theory* (Solt 2008), we expect a positive association between economic inequality and the income gap in voting in relatively wealthy countries. Based on recent studies that included a wider sample of countries (Amat and Beramendi 2016; Matsubayashi and Sakaiya 2018), we expect a negative association between economic inequality and the income gap in voting in less-wealthy countries. Moreover, we investigate whether the prevalence of clientelism is the underlying mechanism that accounts for the presumed negative interaction between relative income and economic inequality at lower levels of national wealth per capita.

We used the Harmonised PolPart^1^ dataset, which combines harmonised survey-data from European Social Survey (ESS), Americas Barometer from Latin American Public Opinion Project (LAPOP), Asian barometer (ASIAN), World Values Study (WVS) and International Social Survey Programme (ISSP).^2^ Our final sample of analysis consists of 66 countries and 292 country-years, including 510,184 individuals. We included a significant number of countries from different continents and thus exceed the most elaborate sample used in previous studies that explicitly tested the relationship between income inequality and voting [i.e. Matsubayashi and Sakaiya (2018): sample of 49 countries and 145 country-years].

## Theory

### Relative Income and Voting

We start at the individual level, with the relationship between an individual’s relative income and voting. In line with previous literature, we focus on an individual’s relative rather than absolute income. Relative income indicates an individual’s economic position, relative to other individuals in his or her country. As Solt (2008) argued, an individual’s inclination to vote does not only depend on how much money he or she has, but also on how much money everyone else has.

From the resource mobilisation perspective (see Brady et al. 1995) it is argued that people need certain resources, like time, money and civic skills to be able to participate in politics. Income can also be regarded as an incentive for political participation (Beramendi and Anderson 2008), indirectly related to voting through attitudes towards politics. Among the most powerful explanations for why richer people are more likely to vote is that people with higher income are more interested in politics, because of what they can gain or lose by (not) having their representatives in political institutions (Gilens 2005). In other words, voting can be efficiently used by the rich for power-orientated (instrumental) reasons. Moreover, financial well-being has been shown to be positively related to levels of political trust (Schäfer 2013; Catterberg and Moreno 2006; Van der Meer and Dekker 2011), and political trust in turn boosts the likelihood of voting because it implies positive expectations towards political institutions (Grönlund and Setälä 2007; Hooghe and Marien 2013). To sum up the theoretical arguments, it is well established in the literature that higher income citizens are more likely to vote compared to lower income citizens.

### Relative Income, Economic Inequality and Voting

Comparative research in relatively advanced and wealthy democracies has indicated that the effect of relative income on voting is even stronger when the level of economic inequality is higher (Gallego 2015; Scervini and Segatti 2012; Solt 2008, 2010; Schäfer and Schwander 2019; but see also Filetti and Janmaat 2018). From the *relative power theory* (see Solt 2008, 2010) it is assumed that more political power is in the hand of the higher income groups when economic inequality is higher. This relatively large advantage allows wealthier citizens to dictate which issues will structure the political debate, and which ones will not be open for deliberation. The higher the economic inequality, the more the political debate will be determined by higher income individuals’ demands. From this perspective, it is thus assumed that economic inequality implies a concentration of not only money but also power and influence in the hands of higher income groups (Gallego 2015). This might explain, according to Solt (2008), why poorer citizens in unequal countries feel even less efficacious and more deprived from the political processes than the poor in less unequal countries. Therefore low income groups are less likely to vote when economic inequality is higher compared to when inequality is lower. Higher income groups are also assumed to be less likely to vote at higher levels of inequality, since the need to defend their interests from the demands of the lower income groups declines when these issues are not salient in the public debate, and when the turnout among the lower income groups is lower. However, according to relative power theory, the higher income groups are still motivated to participate in politics to some extent due to their conflicts with each other. In sum, the *relative power theory* predicts that economic inequality depresses the likelihood to vote for all income groups, but this effect is assumed to be stronger for the lower income groups (Solt 2008).

When especially the lower income groups vote less in more unequal countries, the income gap in voting is larger in these countries, and the effect of income on voting thus is stronger. The above-mentioned studies focused specifically on advanced democracies that are relatively wealthy and thus left the association between economic inequality and voting turnout in less developed countries unexplored. Solt (2008) explicitly left the less developed economies from his study, because he assumed that lower-income citizens in those countries are likely to trade their political support for particularistic benefits.

Indeed, more recent findings from studies that included wealthy as well as less-wealthy countries in their sample show a pattern that contrasts *relative power theory*’s expectations. In a broad sample of countries from various continents, two recent studies concluded that higher levels of economic inequality seem to increase voting turnout of especially the lower income groups, resulting in smaller income gaps in voting in countries with high levels of inequality (Amat and Beramendi 2016; Matsubayashi and Sakaiya 2018). To explain the association between economic inequality and the income gap in voting, Amat and Beramendi (2016) elaborated on the role of *clientelism* as a mobilisation strategy of political parties. When following a clientelistic strategy, parties or politicians provide goods and services targeted to individual or small groups of voters, in exchange for the latter to reciprocate their vote. This exchange may have a short-term character, for example when promising consumables, benefits or marketable goods, but it may also be built around a longer-term relationship, for example when promising access to services or employment over an extended period of time (Kitschelt and Kselman 2013). According to Amat and Beramendi (2016), a clientelistic strategy mobilizes the lower income groups, because lower income citizens are more responsive to direct benefits or bribes compared to higher income citizens. Following Matsubayashi and Sakaiya’s (2018) argument about vote-buying, the likelihood to vote for higher income groups might be negatively affected by clientelistic mobilisation strategies. Since they are not the target of vote-buying, they might perceive political parties that employ clientelistic strategies as corrupt and unfair, which might negatively affect their sense of efficacy and their likelihood to vote (Matsubayashi and Sakaiya 2018).

Comparing the results between the various studies, and the proposed underlying mechanisms, leads us to argue that different mechanisms are at play in wealthy and less-wealthy countries. The association between economic inequality and the income gap in voting thus may depend on levels of national wealth. To test this explicitly, Jensen and Jespersen (2017) analysed data from the European Social Survey and included not only the rich European countries, but also less-wealthy Eastern European countries. They found that in general the positive effect of relative income on voting is *weaker* when economic inequality is higher, but additionally they found that this pattern differs between the wealthier and less-wealthy countries in Europe. In the wealthy countries, the effect of income on voting turnout was stronger when economic inequality was higher, whereas the effect of income was weaker when economic inequality was higher in less-wealthy countries. We aim to test whether this pattern can be generalized to a broader sample of countries, including wealthy and less-wealthy countries from several continents. Therefore we hypothesize that the relationship between economic inequality and the income gap depends on national wealth in the following way:

#### **H1**

The effect of relative income on voting is *stronger* when economic inequality is higher, *in relatively wealthy countries.*

#### **H2**

The effect of relative income on voting is *weaker* when economic inequality is higher, *in less-wealthy countries*.

Next, we focus on the role of clientelism in explaining the different patterns in wealthy and less-wealthy countries. Amat and Beramendi (2016) propose the conditions under which the political elites choose clientelism as a mobilisation strategy. As a first condition, the bureaucratic capacity of the state should be relatively low. In this case political elites have the capacity to hide part of their income from taxes, so that it is easier to use it for clientelistic purposes. Second, the share of low income citizens should be large, making clientelism an attractive strategy for political elites because low income citizens are assumed to be most responsive to this strategy. Third, the income of this group should be low, making the poor extra responsive to bribes or other forms of clientelism. Since national wealth and bureaucratic capacity are very strongly correlated (Kasara and Suryanarayan 2015), we assume that politicians are more likely to choose clientelistic mobilisation strategies when the average wealth in their country is low. Since levels of clientelism are presumably much higher in the less-wealthy countries, we argue that clientelism explains the predicted negative effect of economic inequality on the income gap in voting in these countries (see H2). To test this, we hypothesize:

#### **H3**

The negative interaction between relative income and economic inequality in the less-wealthy countries can be explained by the interaction between relative income and clientelism.

In the wealthier countries levels of clientelism are presumably lower and therefore we expect that clientelism will not cancel out the mechanism proposed in the relative power theory: that with higher economic inequality the lower income groups have less political trust and interest and are therefore less likely to vote.

## Data

To test our hypotheses we used data from the harmonised PolPart dataset^3^ which combines existing survey data on political participation form different cross-national surveys into a single dataset. For the analysis, we used data from multiple waves of ESS,^4^ LAPOP,^5^ ASIAN,^6^ WVS^7^ and ISSP.^8^ Compared to the sample of countries used in previous studies, we especially added non-western countries. These data were collected between 2001 and 2016. A list of all included countries and corresponding survey waves in our sample of analysis is available in the supplementary materials. The individual-level data in the harmonised PolPart dataset were enriched with contextual data on economic inequality and clientelism at the country level, corresponding to the year in which the specific survey wave was performed. After listwise deletion, and after dropping five countries that score lower than 4 on the Freedom House Polity scale to minimize the risk of including countries where elections are very unfree, our final sample of analysis consists of 66 countries and 292 country-year combinations, and a total number of 510,184 respondents. Due to the large number of respondents from a large number of countries, representing six continents, the dataset allows us to study the income gap in voting at a wide range of levels of economic inequality, national wealth and clientelism.

### Measurement

All individual-level variables that were used in the analyses were harmonised before they were included in the PolPart dataset. See Table 1 for the descriptive statistics of all variables. See Online Appendix 2 for a detailed description of the harmonisation procedure for each variable.

**Table 1:** Descriptive statistics for all variables in our sample of analysis

	Mean	SD	Min	Max
*Individual level (N* = *510,184)*				
Voting	0.76		0	1
Relative income	2.71	1.37	1	5
Education	2.94	1.37	1	5
Age	45.95	17.56	15	110
Female	0.52		0	1
*Country-year level (N* = *292)*				
Gini coefficient	0.35	0.08	0.23	0.53
GDP per capita	26.47	15.04	1.17	74.78
Clientelism	0.25	0.23	0.02	0.89
Compulsory voting	0.29		0	1
Majoritarian electoral system	0.10		0	1
Proportional electoral system	0.68		0	1
Mixed electoral system	0.22		0	1

#### Dependent Variables

*Voting* In all surveys that were included in the harmonised PolPart dataset, respondents were asked whether they had voted in the last (national, parliamentary or presidential) election. In the harmonisation procedure all respondents’ answers were recoded into two categories: yes (1) and no (0).

#### Independent Variables

##### Relative Income

Household income was measured differently across surveys. Some surveys measured relative income, operationalised with income deciles (ESS after 2006) or quintiles (ASIAN, ISSP). The original deciles or quintiles from these surveys are based on the actual income distribution in the countries. Other surveys (LAPOP, WVS, ESS before 2006) measured income in a number of income categories, not based on the actual income distribution in the country. Therefore we based the income quintiles for each country-year in these surveys on the distribution of income in the corresponding sample. We recoded the income variables from all surveys into income quintiles, indicating in which of the twenty-percent income groups the respondent’s income falls in the corresponding country: lowest income quintile (1), second (2), third (3), fourth (4), and highest income quintile (5). For a detailed explanation of how the income variable was harmonised across surveys, see Online Appendix 2.

To be sure that differences in the effect of relative income on voting are not caused by differences in measurement across surveys, we did some additional analyses (see Online Appendix 3). We took advantage of the fact that 21 countries are included in the ISSP in the same year that they were also included in one of the other surveys (ESS, LAPOP and ASIAN). We estimated the effect of relative income on voting for each country-year that is included in the ISSP survey and simultaneously in either ESS, LAPOP or ASIAN. Similarly, we found 18 countries that are both included in the ISSP 2004 and/or the WVS 5, and also included in either the ESS, LAPOP or ASIAN. The coefficient plots in Online Appendix 3 show the effects of relative income for each country-year for the surveys in which this country-year is included. In general, the effect of income is similar when compared between surveys within country-years. There are only a few country-years in which we found the effect to be substantially differ between surveys. Altogether, based on these additional analyses we are confident that the differences in the effects of income on voting between country-years are not caused by differences in the operationalization of income.

##### Education

We include individuals’ education in our analyses, as it is considered one of the most important resources for political participation (Dalton 2004). Respondents were asked about the highest level of education they accomplished in all surveys. The number of answer categories varied between the surveys. The education variables were harmonised into a 5-category variable with categories: No education or only primary education (1); Some or lower secondary education (2); Completed or higher secondary education (3); Some (non-university) tertiary education (4); and University (5).

##### Female

To control for a respondent’s gender we included a dummy variable that indicates whether a respondent is female (1) or male (0).

##### Age

To control for a respondent’s age we included a continuous variable that measures the absolute age of a respondent in years. We also created a quadratic term and included this in our models to account for possible non-linear effects of age.

##### Economic Inequality

To measure economic inequality at the contextual level we used the Standardized World Income Inequality Database (SWIID). This dataset was constructed based on data from many national and international data sources. The SWIID uses a missing data multiple imputation algorithm to standardize the observations that were collected from the various source datasets in order maximize the comparability of Gini coefficients for the broadest possible sample of countries and years (Solt 2016). This means that all Gini coefficients are estimated 100 times for each observation (i.e. each country-year combination) in the SWIID dataset to capture the uncertainty in the estimates. We computed the Gini coefficient by taking the mean of the 100 estimates for each observation. We used the Gini index of net income inequality, which measures the distribution of household income after government taxes and transfers. A score of 0 indicates that each household receives an equal share of the income in the country, and 1 indicates that all income is received by a single household. For easier interpretation of interaction effects, we centred this computed Gini coefficient around the mean at the country-year level in the sample of analysis.

##### Wealth

Data about the countries’ GDP per capita were retrieved from the Penn World Table (PWT) dataset (see Feenstra et al. 2015). For easier interpretation of interaction effects, we centred this variable around the mean at the country-year level in the sample of analysis.

##### Clientelism

To measure the level of clientelism at the contextual level, we used the clientelism index from the Varieties of Democracy (V-Dem) dataset^9^ (Sigman and Lindberg 2017). It measures to what extent politics is based on clientelistic relationships. The index is formed by combining three components. The first component is vote-buying, measuring the extent to which vote buying was common during the most recent national election in the country. The second component is the extent to which social expenditures in the national budget are particularistic (i.e. narrowly targeted on a specific corporation, sector, social group, region, party, or set of constituents) as opposed to public good expenditures. The third component is party linkages, which refers to what sort of goods political parties offer in exchange for political support (clientelistic vs. programmatic). A high score on this clientelism index reflects that politics is to a large extent based on clientelistic relationships. For easier interpretation of interaction effects, we centred the clientelism index around the mean at the country-year level in the sample of analysis.

##### Compulsory Voting

We used the database from the International Institute for Democracy and Electoral Assistance (IDEA)^10^ to control for whether voting is compulsory (1) or not (0).

##### Electoral System

To account for differences in electoral systems we used the ‘Electoral System Type’ variable from the V-Dem dataset, which distinguishes between proportional, majoritarian and mixed electoral systems.

## Analytical Strategy

Before we tested our hypotheses, we did some exploratory analyses at the country-year level, in which we plotted the income gap in voting against economic inequality. To formally test our hypotheses, we performed multilevel logistic regression models with three levels of analysis in order to correct the standard errors for the nested structure of our data, with individuals nested in country-years, nested in countries. Ignoring the multilevel structure in the data would have led to underestimation of the standard errors (Steenbergen and Jones 2002). As a first step, we performed a model with the main effects of relative income, the individual-level control variables, and economic inequality and national wealth as independent variables. In the second step, we tested whether the effects of relative income on voting varied with the level of economic inequality, by adding a cross-level interactions between relative income and economic inequality to the previous model. Since we expected that this cross-level interaction effect is negative in the less-wealthy countries and positive in the wealthy countries, we did not have a hypothesis for this interaction in the complete sample of countries. In the third step, we tested the three-way interaction between relative income, economic inequality and national wealth, to test Hypotheses 1 and 2.

Based on the results of these analyses we proceeded to the fourth step in order to test Hypothesis 3. Although we expected to find a negative interaction between economic inequality and relative income on voting only in less-wealthy countries, our results showed that this negative interaction holds in the complete sample and does not vary with levels of national wealth. We examined whether this can be explained by including clientelism. That is, we examined whether including a cross-level interaction between relative income and clientelism can explain the negative cross-level interaction between relative income and economic inequality.

## Results

### Exploratory Analyses

**Fig. 1:**
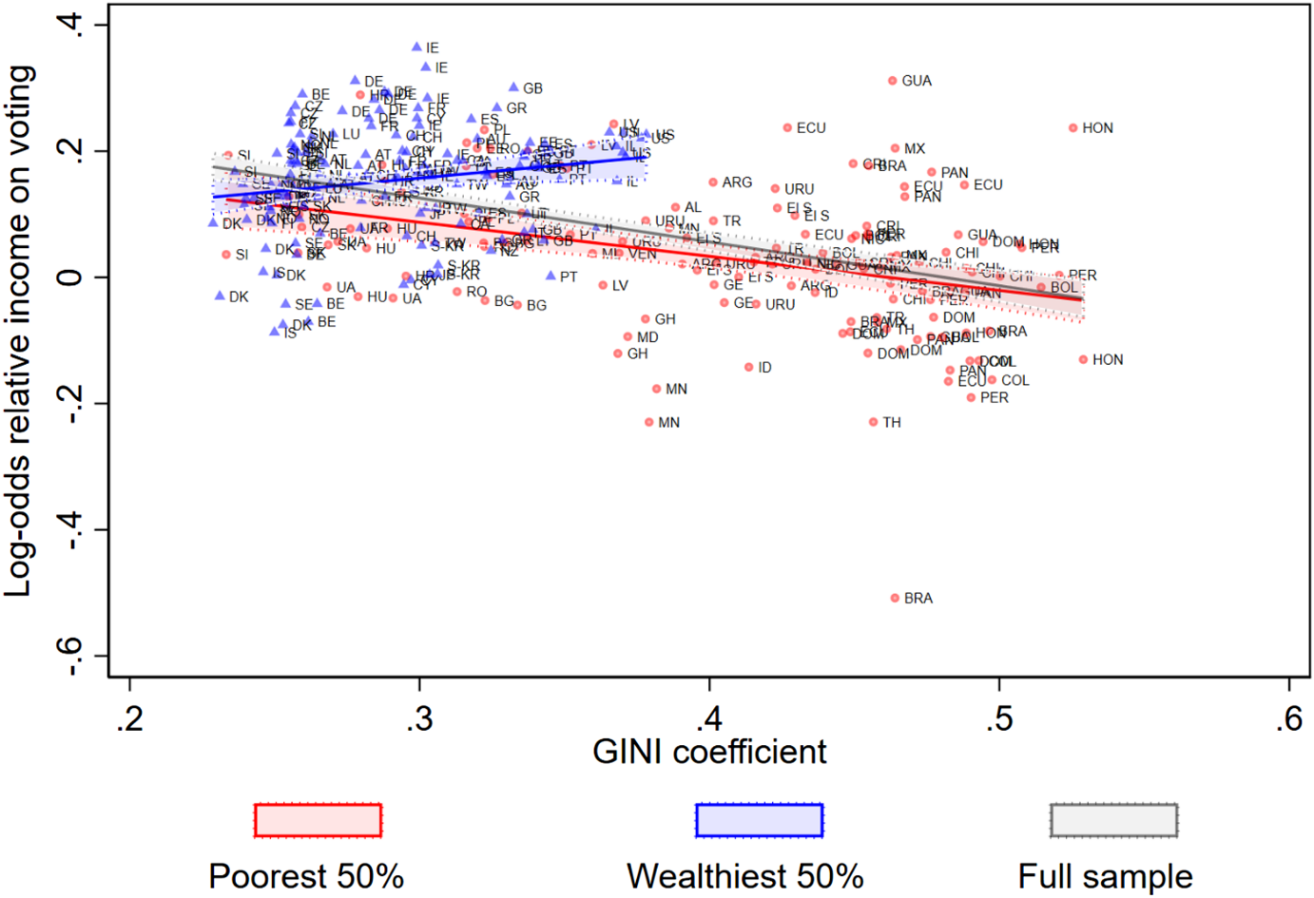
Logistic regression coefficient for the effect of relative income on voting (controlled for education, gender and age) as a function of economic inequality (Gini coefficient) at the country-year level,, with regression lines fitted for the full sample, the poorest 50% and the wealthiest 50% country-years

First, we ran a series of logistic regression models to test the effect of relative income on voting in each separate country-year subsample in our data. We controlled for age, gender and education. Subsequently, we plotted the resulting regression coefficients (the log-odds), as an indicator of the income gap in voting, against economic inequality in Fig. 1. The figure shows that in most countries there is a ‘positive income gap’ in voting; individuals from higher income groups are more likely to vote than individuals from lower income countries, yet in a substantial number of countries there is a ‘negative income gap’. We fitted a regression line through the whole sample of country-years, and separate regression lines for the 50% poorest country-years and the 50% wealthiest country-years (based on GDP p.c.). Figure 1 shows that economic inequality is, overall, negatively related to the income gap in voting (r = − 0. 478, p < 0.001). However, as we expected, the association seems to depend on levels of national wealth. In the 50% poorest country-years, we see a negative association between economic inequality and the income gap in voting (r = − 0.354, p < 0.001). In the group of wealthier countries this association is slightly positive (r = . 183, p = 0.102).

Second, we explored the bivariate relationships between clientelism on the one hand and inequality, national wealth and the income gap in voting on the other hand. As expected, we found that clientelism is positively related to economic inequality (r = 0.698, p < 0.001), negatively related to national wealth (r = − 0.744, p < 0.001), and negatively related to the income gap in voting (r = − 0.495, p < 0.001) at the country-year level. In the next section we examine if the results of these exploratory analyses hold, by estimating multilevel models with the effect of relative income on voting, and its moderators, including control variables at the contextual level.

### Multilevel Analyses

In Tables 2 and 3 the results of the multilevel analyses are presented. Model 1 of Table 2 contains only the main effects and shows that citizens with a higher relative income are more likely to vote (b = 0.010, se = 0.002), when controlled for contextual-level variables, survey dummies and the individual-level variables age, gender and education. Further, economic inequality is negatively related to the likelihood to vote (b = 0.613, se = 0.235), and so is GDP p.c. (b = − 0.003, se = 0.001). In Model 2 we added the interaction between relative income and economic inequality. The results show that citizens with a higher relative income are more likely to vote (b = 0.079, se = 0.011) at mean levels of economic inequality. This positive effect is weaker at higher levels of economic inequality (b _relative income × Gini_ = − 0.693, se = 0.133). This indicates that the income gap in voting is smaller at higher levels of economic inequality and may eventually turn into a ‘negative income gap’.

**Table 2:** Results of the multilevel regression models on voting, including the moderating effects of economic inequality and national wealth (N_countries_ = 66; N_country-years_ = 292; N_individuals_ = 510,184)

	Model 1		Model 2		Model 3		Model 4	
	*B*	*SE*	*B*	*SE*	*B*	*SE*	*B*	*SE*
Income	0.010***	0.002	0.079***	0.011	0.088***	0.014	0.083***	0.014
Gini	− 0.613**	0.235	− 2.840	1.546	− 5.675**	1.822	− 5.420**	0.218
GDP p.c	− 0.003**	0.001	− 0.019**	0.006	− 0.029***	0.007	− 0.289***	0.008
Income × Gini			− 0.693***	0.133	− 0.327	0.205	− 0.586**	0.218
Income × GDP p.c					0.003**	0.001	0.004**	0.001
Gini × GDP p.c					− 0.179*	0.079	− 0.207	0.109
Income × Gini × GDP p.c					0.008	0.011	0.012	0.014
GDP p.c.^2^							0.000	0.000
Income × GDP p.c.^2^							0.000	0.000
Gini × GDP p.c.^2^							− 0.003	0.004
Income × Gini × GDP p.c.^2^							0.001	0.001
*Control variables*								
Education	0.035***	0.000	0.238***	0.003	0.238***	0.003	0.238***	0.003
Age	0.027***	0.000	0.149***	0.001	0.149***	0.001	0.149***	0.001
Age squared	− 0.000***	0.000	− 0.001***	0.000	− 0.001***	0.000	− 0.001***	0.000
Female	0.001	0.001	− 0.008	0.007	− 0.008	0.007	− 0.008	0.007
Clientelism	0.029	0.077	− 0.046	0.506				
Comp. voting	0.085*	0.037	0.518*	0.251	0.547*	0.246	0.531*	0.253
*Elect. syst. (Major.* = *ref.)*								
Proportional	− 0.026	0.040	− 0.172	0.271	− 0.285	0.272	− 0.309	0.281
Mixed	− 0.005	0.041	0.007	0.274	− 0.069	0.270	− 0.100	0.282
*Survey (ESS* = *ref.)*								
WVS	0.072***	0.003	0.499***	0.020	0.499***	0.020	0.499***	0.020
LAPOP	0.177***	0.005	1.187***	0.036	1.191***	0.036	1.191***	0.036
ASIAN	0.027***	0.008	0.145*	0.058	0.147*	0.058	0.149**	0.058
ISSP	− 0.000	0.002	0.009	0.016	0.010	0.016	0.010	0.016
Intercept	− 0.155***	0.037	− 3.966***	0.252	− 4.047***	0.251	− 3.977***	0.275
*Variance components*								
Country								
RE income	0.000	0.000	0.006	0.001	0.005	0.001	0.005	0.001
Intercept	0.012	0.002	0.606	0.119	0.584	0.114	0.578	0.114
Country-year								
RE income	0.000	0.000	0.004	0.000	0.004	0.001	0.004	0.001
Intercept	0.003	0.000	0.128	0.014	0.126	0.014	0.126	0.014

Gini, Clientelism and GDP p.c. are centered around the mean at the country-year level

*Ref* reference category, *Comp. voting* compulsory voting, *Elect. syst.* electoral system, *GDP p.c.*^2^ quadratic GDP p.c.

*p < 0.05; **p < 0.01; ***p < 0.001

**Table 3:** Results of the multilevel regression models on voting, additionally including the moderating effect of clientelism (N_countries_ = 66; N_country-years_ = 292; N_individuals_ = 510,184)

	Model 5		Model 6		Model 7	
	B	SE	B	SE	B	SE
Income	0.083***	0.011	0.077***	0.016	0.082***	0.010
Clientelism	0.111	0.505	− 0.178	0.696	0.045	0.506
GDP p.c	− 0.020**	0.006	− 0.016*	0.008	− 0.019**	0.006
Gini	− 3.356*	0.537			− 3.060*	(0.544
Income × Client	− 0.237***	0.046	− 0.169	0.091	− 0.147**	0.057
Income × GDP p.c			0.002	0.001		
Client. × GDP p.c			0.011	0.033		
Income × Client. × GDP p.c			− 0.002	0.004		
Income × Gini					− 0.419*	0.167
*Control variables*						
Education	0.238***	0.003	0.238***	0.003	0.238***	0.003
Age	0.149***	0.001	0.149***	0.001	0.149***	0.001
Age squared	− 0.001***	0.000	− 0.001***	0.000	− 0.001***	0.000
Female	− 0.008	0.007	− 0.008	0.007	− 0.008	0.007
Compulsory voting	0.538*	0.250	0.286	0.232	0.526*	0.250
*Elect. syst. (Major.* = *ref.)*						
Proportional	− 0.183	0.271	− 0.126	0.277	− 0.179	0.270
Mixed	− 0.005	0.273	0.029	0.284	− 0.001	0.273
*Survey (ESS* = *ref.)*						
WVS	0.499***	0.020	0.498***	0.020	0.499***	0.020
LAPOP	1.188***	0.036	1.187***	0.036	1.187***	0.036
ASIAN	0.146*	0.058	0.145*	0.058	0.146*	0.058
ISSP	0.009	0.016	0.009	0.016	0.009	0.016
Intercept	− 3.964***	0.251	− 3.897***	0.283	− 3.963***	0.251
*Variance components*						
*Country*						
RE income	0.006	0.001	0.006	0.001	0.006	0.001
Intercept	0.602	0.118	0.651	0.126	0.601	0.118
*Country-year*						
RE income	0.004	0.001	0.004	0.001	0.004	0.001
Intercept	0.128	0.014	0.128	0.014	0.128	0.014

Gini, Clientelism and GDP p.c. are centered around the mean at the country-year level

*Ref* reference category, *RE* random effect, *Client.* Clientelism, *Comp. voting* compulsory voting, *Elect. syst.* electoral system

*p < 0.05; **p < 0.01; ***p < 0.001

Now that we have analysed the moderating effect of economic inequality, we turn to the question whether the moderating effect of economic inequality varies with levels of national wealth. Model 3 shows that it does not vary with national wealth (b _relative income × Gini × GDP. p.c._ = 0.008, se = 0.011), also not when the interaction is modelled with a quadratic GDP p.c. variable in Model 4. We included the quadratic GDP p.c. variable because the effect of GINI on the income gap in voting might depend on GDP p.c. in a non-linear way. Hypothesis 2 is therefore only partly supported, since we expected to find a negative interaction between relative income and economic inequality in less-wealthy countries. However, the results suggest that this pattern is present in the complete sample of country-years. Hypothesis 1, which predicted a positive effect of economic inequality on the income gap in voting in wealthy countries, is thus rejected by these results. The results of our bivariate analyses, where we found differences between more- and less-wealthy countries, do not hold after employing multilevel logistic regression models with contextual-level control variables.

Subsequently, because we found a negative interaction between economic inequality and income in our complete sample, we examined the role of clientelism in our complete sample, instead of only in less-wealthy countries. First, we tested whether the effect of relative income is weaker at higher levels of clientelism. Second, we examined whether this in turn varies with levels of national wealth, since we initially anticipated that clientelism would be especially prevalent in less-wealthy countries. Third, we analysed whether clientelism (partly) explains the negative interaction between relative income and economic inequality. The results of Model 5 in Table 3 show that relative income is positively related to voting (b = 0.083, se = 0.011) at mean levels of clientelism, and that this effect is weaker at higher levels of clientelism (b _relative income × clientelism_ = − 0.237, se = 0.046). The results of Model 6 show that the interaction between relative income and clientelism does not vary with levels of national wealth per capita (b_relative income × Clientel × GDP. p.c._ = − 0.002, se = 0.004).

**Fig. 2:**
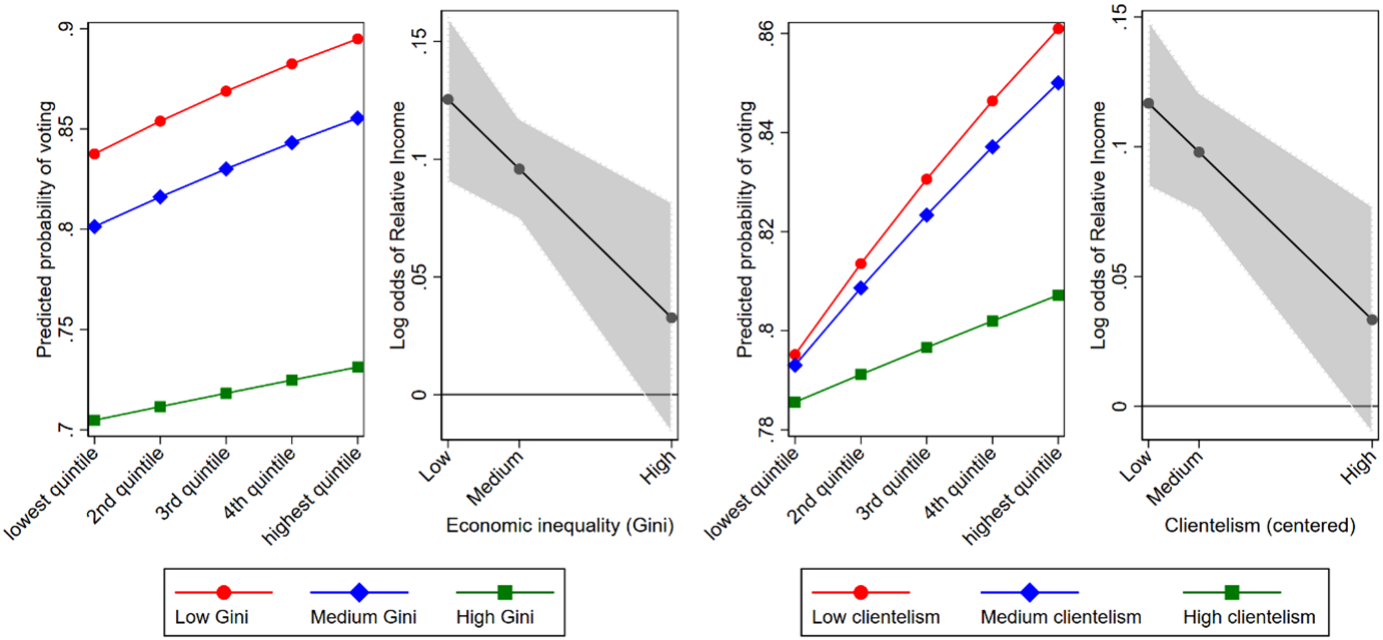
Predicted probabilities of voting by income quintile, and the marginal effects of relative income on voting, as a function of economic inequality (left panel) and clientelism (right panel) based on Model 7. *Note* Model 7 includes the interaction between relative income and economic inequality as well as the interaction between relative income and clientelism. The 10th percentile in the distribution of economic inequality (Gini) and clientelism at the country-year level was taken as the low level. The 50th percentile was taken as the medium level, and the 90th percentile was taken as the high level

The results of Model 7 show that the interaction between relative income and clientelism cannot completely explain the negative interaction between relative income and economic inequality that we found in Model 2. Namely, the interaction between relative income and clientelism is again significant (b_relative income × clientelism_ = − 0.147, se = 0.057) when both interaction terms are included, but the interaction between relative income and inequality is also still significant (b _relative income × Gini_ = − 0.419, se = 0.167). Including the interaction between relative income and clientelism decreased the moderating effect of economic inequality with about 40% compared to Model 2. Figure 2 shows the predicted probabilities of voting for all income quintiles, and the marginal effects of relative income, at different levels of inequality (left panel) and clientelism (right panel) based on Model 7.

The left panel of Fig. 2 shows that the likelihood to vote decreases for all income groups when economic inequality is higher. This effect is slightly stronger for the higher income groups, and therefore the income gap in voting is smaller when economic inequality is higher. The right panel of Fig. 2 shows that the decreasing effect of income at higher levels of clientelism is due to the *higher* income groups being *less* likely to vote. This is in line with the argument that clientelism negatively affects the sense of efficacy and the likelihood to vote for higher income groups. However, the likelihood to vote for the *lower* income groups is hardly affected by the level of clientelism. This is in *not* line with our argument that the lower income groups are mobilized when political parties use clientelistic mobilisation strategies. In sum, the prevalence of clientelism may be one of the mechanisms through which economic inequality negatively affects the income gap in voting, but it does not affect lower income groups in the way we anticipated.

## Conclusions and Discussion

In this study we investigated whether the income gap in voting turnout varies with economic inequality at the country-year level. We aimed to bring together previous studies on economic inequality and the income gap in voting, since the results of these studies contradict each other. We did so by formulating new hypotheses. First, we expected the relationship between economic inequality and the income gap in voting to vary by the national level of wealth. Based on the *relative power theory* (Solt 2008), we expected a positive association between economic inequality and the income gap in voting in relatively wealthy countries (H1). Based on recent studies that included a wider sample of countries (Amat and Beramendi 2016; Matsubayashi and Sakaiya 2018), we expected a negative association between economic inequality and the income gap in voting in less-wealthy countries (H2). Second we hypothesized that clientelism would explain the negative interaction between income and inequality at lower levels of national wealth. Namely, we anticipated that the prevalence of clientelism would increase the likelihood to vote for lower income groups, decrease the likelihood to vote for higher income groups, and therewith weaken the positive effect of income on voting. Our final sample of analysis consists of 68 countries from different continents, and 292 country-years, including 510,184 individuals, and thus exceed the most elaborate sample used in previous studies that explicitly tested the relationship between income inequality and voting.

We found that, in general, higher income groups are more likely to vote. The positive effect of income was found to be weaker at higher levels of economic inequality. We found that this pattern does not vary with levels of national wealth, which means that Hypothesis 1 is not supported. This finding does not support our predictions based on *relative power theory.* This does not imply that we did not find any evidence for the relative power theory; the negative main effect of economic inequality on voting is in line with this theory, however, the theory’s prediction that increasing economic inequality also results in an increased income gap in voting is not supported. The latter is not in line with conclusions from Schäfer and Schwander’s (2019) recent work in which they studied 21 OECD countries. They controlled for a wider variety of country(-year) characteristics, their data covered a longer time period but a smaller sample of countries, and the individual-level control variables differ from our models. For example, they included union membership and marital status, but not education. Moreover, it has to be noted that the positive interaction between relative income and economic inequality that Solt (2008) described was in fact not significant, in Gallego’s (2015) study it was gross income inequality that predicted a larger income gap in voting, whereas no effect of net income inequality was found, and Filetti and Janmaat (2018) also found no significant interaction when it comes to voting. When combining our results with those of earlier studies, it seems that altogether there is inconclusive evidence about whether higher net income inequality is related to a higher income gap in turnout in wealthy countries. Future studies should therefore still try to uncover the presumed mechanism through which economic inequality would widen the income gap in voting more precisely, and the conditions under which this would occur, in order to shed more light on the inconclusive findings in the literature so far.

Although our results do not underline the concerns based on *relative power theory*’s predictions, i.e. political and economic inequality reinforce each other, our findings support previous studies’ conclusions that voter turnout in general is depressed by economic inequality (Horn 2011; Polacko et al. 2020; Solt 2008; but see Stockemer and Scruggs 2012), which raises other concerns. Low rates of participation may be regarded as a sign of disaffection and a threat to the functioning of democracy (Lijphart 1997; Norris 1999; but see Rosema 2007; Lutz and Marsh 2007). Moreover, that the income gap in *voting* does not vary with economic inequality in wealthier countries does not necessarily mean that political inequality remains unaffected. Higher socioeconomic groups participate to a higher rate in other forms of politics as well (e.g. Brady et al. 1995; Hakhverdian et al. 2012), and Solt (2015) showed that relative power theory’s predictions are confirmed for various forms of non-violent protest. Similarly, Filetti and Janmaat (2018) showed that in Europe, income inequality widens the gap between the rich and the poor for boycotting products and signing petitions, but not for voting. Moreover, political inequality is more than inequality in political participation. It also comprises the extent to which various groups are involved in civil society organizations, are represented in government, are able to set the political agenda, and influence political decisions and the implementations of those decisions (Houle 2018; Schakel 2020). When taking such a broad conceptualization of political equality, it was found to be negatively related to economic inequality at the macro level in a global sample of countries (Houle 2018). Moreover, policy in advanced democracies is more responsive to higher income groups than it is to middle- and low-income groups (Schakel 2020). Altogether, our results combined with other studies do not imply that economic and political inequality cannot reinforce each other, but it is important to study their interrelationships for various forms of political participation and other forms of power, to get a better insight in how economic inequality affects political inequality, and vice versa, in wealthy democracies.

The negative association between economic inequality and the income gap in voting is in line with Hypothesis 2 for the less-wealthy countries. However, since we found this pattern in the complete sample of countries, we investigated the moderating role of clientelism in the complete sample. These analyses showed that the effect of relative income is weaker when levels of clientelism are higher, which was constant across levels of national wealth. When clientelism and economic inequality were both included as moderators, both interactions were significant, but the interaction between economic inequality and income was reduced. It suggests that although clientelism might partially explain why economic inequality reduces the income gap in voter turnout, there must also be other mechanisms that explain a negative relationship between economic inequality and political inequality. Hypothesis 3 was therefore only partly supported by our results.

Moreover, the prevalence of clientelism does not exactly work as we argued. The lower income groups are *not* more likely to vote when clientelism is higher, which is not in line with Amat and Beramendi’s (2016) argument that lower income groups would be responsive to direct short-term benefits. However, higher income groups are less likely to vote when clientelism is higher, which is in line with the theoretical mechanism we outlined based on Matsubayashi and Sakaiya’s (2018) argument on vote-buying. Since higher income citizens are not the target of clientelistic strategies, they might perceive political parties that employ such strategies as corrupt and unfair, negatively affecting their sense of political trust and efficacy, and their likelihood to vote. Since our results suggest that especially the higher income groups are responsive to contextual conditions, further studies should analyze more precisely why, beyond only focusing on political parties’ mobilization strategies. Following Kasara and Suryanarayan (2015, 2020), higher income groups are more likely to vote when the potential tax exposure of the rich is high. This is the case when the state’s capacity to tax income is higher. It is likely that the poorer, more unequal countries with high levels of clientelism in our sample are also countries with low bureaucratic capacity (Kitschelt and Wilkinson 2007; Kasara and Suryanarayan 2015). Future studies could therefore explore to what extent the associations between clientelism and inequality on the one hand, and the income gap in voting on the other hand, can be explained by the state’s bureaucratic capacity and government effectiveness (Kasara and Suryanarayan 2020).

This study was limited in certain aspects and therefore future research could try to improve upon our study in several other ways. First, we only looked at the effect of net income inequality measured with the Gini coefficient. Future studies may compare different measures of economic inequality (e.g. gross Gini, P90/P10) to study whether our results are robust to different operationalizations of economic inequality (see Gallego 2015 for a discussion on gross and net inequality). Second, our measure of relative income is not perfectly comparable between surveys, although we created income quintiles for each country-year for each survey. Namely, in some surveys these quintiles were based on the actual income distribution in the country (ESS after 2006, ASIAN & ISSP) and in some surveys these categories were based on the income distribution of the sample (LAPOP, WVS, ESS before 2006). We explored the extent to which the differences in measurement between surveys affected the estimate for the effect of relative income on voting in Online Appendix 3. These comparisons lead us to cautiously conclude that, overall, differences in the effect of income are not caused by different operationalizations of the income variable between surveys. To control for survey differences in our models, we included dummy variables for the surveys in each model.

Notwithstanding these limitations, this study adds some important insights to the literature on economic and political inequality. Our study’s results question the assumption that economic inequality undermines political equality in voting in wealthy democracies and indicate that, in general, economic inequality is even negatively related to inequality in voting. Although clientelism partially explains why economic inequality reduces the income gap in voter turnout, it does not do so in the way we expected. Importantly, our results do not imply that economic inequality is positive for democratic representation, since economic inequality was found to depress the likelihood of voting for all income groups, and other studies indicated that economic inequality is associated with political inequality in several other ways than through voting.

## Electronic supplementary material

Below is the link to the electronic supplementary material.

Supplementary file1 (DOCX 22 kb)

Supplementary file2 (DOCX 32 kb)

Supplementary file3 (DOCX 1198 kb)

## Data Availability

Instructions for reconstructing the dataset can be found at Open Science Framework via: https://osf.io/gztky/. Codes for replicating the analyses can be found at Open Science Framework via: https://osf.io/gztky/.

## Footnotes

1 This study was conducted within the PolPart project funded by ERC Grant No. 339829. The full title of the project reads ‘How citizens try to influence politics and why? International comparisons of movement and party politics’. Principal Investigator is Prof. Dr. Bert Klandermans.

2 Unfortunately, we could not include data from the African Barometer in our analyses, since no comparable measure of (relative) income is available from this survey.

3 Instructions and codes for reconstructing this dataset, and for replicating our analyses, can be found via the Open Science Framework page: https://osf.io/gztky/.

4 *Source*: European Social Survey Cumulative File, ESS 1–7 (2016). Data file edition 1.0. NSD—Norwegian Centre for Research Data, Norway—Data Archive and distributor of ESS data for ESS ERIC. ESS Round 8: European Social Survey (2018): ESS-8 2016 Documentation Report. Edition 2.1. Bergen, European Social Survey Data Archive, NSD—Norwegian Centre for Research Data for ESS ERIC.

5 *Source*: The AmericasBarometer by the Latin American Public Opinion Project (LAPOP), www.LapopSurveys.org.

6 *Source*: Asian Barometer. (2018). Asian Barometer Survey Data Release: 2001–2003, 2005–2008, 2010–2012, 2014–2016. https://www.asianbarometer.org.

7 *Source*: Inglehart, R., C. Haerpfer, A. Moreno, C. Welzel, K. Kizilova, J. Diez-Medrano, M. Lagos, P. Norris, E. Ponarin & B. Puranen et al. (eds.). 2014. World Values Survey: Round Five—Country-Pooled Datafile. Version: www.worldvaluessurvey.org/WVSDocumentationWV5.jsp. Madrid: JD Systems Institute.

8 *Source*: ISSP Research Group (2012): International Social Survey Programme 2004: Citizenship I. (ISSP 2004). GESIS Data Archive, Cologne. ZA3950 Data file Version 1.3.0, https://doi.org/10.4232/1.11372. ISSP Research Group (2016): International Social Survey Programme: Citizenship II – ISSP 2014. GESIS Data Archive, Cologne. ZA6670 Data file Version 2.0.0. 10.4232/1.12590.

9 Coppedge, Michael, John Gerring, Carl Henrik Knutsen, Staffan I. Lindberg, Svend-Erik Skaaning, Jan Teorell, David Altman, Michael Bernhard, Agnes Cornell, M. Steven Fish, Haakon Gjerløw, Adam Glynn, Allen Hicken, Joshua Krusell, Anna Lührmann, Kyle L. Marquardt, Kelly McMann, Valeriya Mechkova, Moa Olin, Pamela Paxton, Daniel Pemstein, Brigitte Seim, Rachel Sigman, Jeffrey Staton, Aksel Sundtröm, Eitan Tzelgov, Luca Uberti, Yi-ting Wang, Tore Wig, and Daniel Ziblatt. 2018. “V-Dem Codebook v8” Varieties of Democracy (V-Dem) Project.

10 https://www.idea.int/data-tools/data/voter-turnout/compulsory-voting.

## References

[CR1] Amat, F., & Beramendi, P. (2016). Economic and political inequality: The role of political mobilization. CAGE Online Working Paper Series 277, Competitive Advantage in the Global Economy (CAGE). Retrieved from https://ideas.repec.org/p/cge/wacage/277.html.

[CR2] Armingeon K, Schädel L (2015). Social inequality in political participation: The dark sides of individualisation. West European Politics.

[CR3] Beramendi P, Anderson CJ, Anderson CJ, Beramendi P (2008). Income, inequality, and electoral participation. Democracy, inequality, and representation. A comparative perspective.

[CR4] Brady HE, Verba S, Schlozman KL (1995). Beyond SES: A resource model of political participation. American Political Science Review.

[CR5] Catterberg G, Moreno A (2006). The individual bases of political trust: Trends in new and established democracies. International Journal of Public Opinion Research.

[CR6] Dalton RJ (2004). Democratic challenges, democratic choices: The erosion of political support in advanced industrial democracies.

[CR7] Feenstra RC, Inklaar R, Timmer MP (2015). The next generation of the Penn World Table. American Economic Review.

[CR8] Filetti A, Janmaat JG (2018). Income inequality and economic downturn in Europe: A multilevel analysis of their consequences for political participation. Acta Politica.

[CR9] Gallego A (2015). Unequal political participation worldwide.

[CR10] Geys B (2006). Explaining voter turnout: A review of aggregate-level research. Electoral Studies.

[CR11] Gilens M (2005). Inequality and democratic responsiveness. Public Opinion Quarterly.

[CR12] Grönlund K, Setälä M (2007). Political trust, satisfaction and voter turnout. Comparative European Politics.

[CR13] Hakhverdian A, Van Der Brug W, De Vries C (2012). The emergence of a ‘diploma democracy’? The political education gap in the Netherlands, 1971–2010. Acta Politica.

[CR14] Hooghe M, Marien S (2013). A comparative analysis of the relation between political trust and forms of political participation in Europe. European Societies.

[CR15] Horn, D. (2011). Income inequality and voter turnout. *GINI Discussion Papers*, 16. Amsterdam: AIAS.

[CR16] Houle C (2018). Does economic inequality breed political inequality?. Democratization.

[CR17] Jaime-Castillo AM (2009). Economic inequality and electoral participation: A cross-country evaluation. SSRN Electronic Journal.

[CR18] Jensen C, Jespersen BB (2017). To have or not to have: Effects of economic inequality on turnout in European democracies. Electoral Studies.

[CR19] Kasara K, Suryanarayan P (2015). When do the rich vote less than the poor and why? Explaining turnout inequality across the world. American Journal of Political Science.

[CR20] Kasara K, Suryanarayan P (2020). Bureaucratic capacity and class voting: Evidence from across the world and the United States. The Journal of Politics.

[CR21] Kitschelt H, Kselman DM (2013). Economic development, democratic experience, and political parties’ linkage strategies. Comparative Political Studies.

[CR22] Kitschelt H, Wilkinson S, Kitschelt H, Wilkinson S (2007). Citizen politician linkages: An introduction. Patrons, clients, and policies: Patterns of democratic accountability and political competition.

[CR23] Laurison D (2016). Social class and political engagement in the United States. Sociology Compass.

[CR24] Lijphart A (1997). Unequal participation: Democracy's unresolved dilemma presidential address, American political science association, 1996. American Political Science Review.

[CR25] Lutz G, Marsh M (2007). Introduction: consequences of low turnout. Electoral Studies.

[CR26] Matsubayashi, T., & Sakaiya, S. (2018). Income inequality and voter turnout. Retrieved from https://ssrn.com/abstract=3126657.

[CR27] Norris P (1999). Critical citizens: Global support for democratic government.

[CR28] Polacko M, Heath O, Lewis-Beck M, Dassonneville R (2020). Policy polarization, income inequality and turnout. Political Studies.

[CR29] Rosema M (2007). Low turnout: Threat to democracy or blessing in disguise? Consequences of citizens’ varying tendencies to vote. Electoral Studies.

[CR30] Scervini F, Segatti P (2012). Education, inequality and electoral participation. Research in Social Stratification and Mobility.

[CR31] Schäfer A, Keil SI, Gabriel OW (2013). Affluence, inequality and satisfaction with democracy. Society and democracy in Europe.

[CR32] Schäfer A, Schwander H (2019). ‘Don’t play if you can’t win’: Does economic inequality undermine political equality?. European Political Science Review.

[CR33] Schakel W (2020). Representing the rich: Economic and political inequality in established democracies.

[CR34] Smets K, Van Ham C (2013). The embarrassment of riches? A meta-analysis of individual-level research on voter turnout. Electoral Studies.

[CR35] Sigman, R., & Lindberg, S. I. (2017). Neopatrimonialism and democracy: An empirical investigation of Africa’s Political Regimes. *V-Dem Working Paper Series, 2017 (56)*. Retrieved from www.v-dem.net.

[CR36] Solt F (2008). Economic inequality and democratic political engagement. American Journal of Political Science.

[CR37] Solt F (2010). Does economic inequality depress electoral participation? Testing the Schattschneider hypothesis. Political Behavior.

[CR38] Solt F (2015). Economic inequality and nonviolent protest. Social Science Quarterly.

[CR39] Solt F (2016). The standardized World Income Inequality Database. Social Science Quarterly.

[CR40] Steenbergen MR, Jones BS (2002). Modeling multilevel data structures. American Journal of Political Science.

[CR41] Stockemer D, Scruggs L (2012). Income inequality, development and electoral turnout—New evidence on a burgeoning debate. Electoral Studies.

[CR42] Van der Meer T, Dekker P, Zmerli S, Hooghe M (2011). Trustworthy states, trusting citizens? A multilevel study into objective and subjective determinants of political trust. Political trust: Why context matters.

