# Supplementary material for: The Income Gap in Voting: Moderating Effects of Income Inequality and Clientelism
Source: Polit Behav. 2020 Oct 12;44(3):1203–23. doi: 10.1007/s11109-020-09652-z (PMC9433354; doi:10.1007/s11109-020-09652-z)
Supplement: Supplementary file 1 — Supplementary file1 (DOCX 22 kb) [file 11109_2020_9652_MOESM1_ESM.docx]

**Appendix 1. Overview of all country-years included in our sample**

| **Country** | **Years and surveys** | |
| --- | --- | --- |
| BG | 2004^a e^ 2006^b^ | |
| RO | 2004 ^a^ | |
| TR | 2004 ^a^ | |
| UA | 2004 ^a^ 2008 ^b^ 2010 ^b^ 2012 ^b^ | |
| AL | 2012 ^b^ | |
| GE | 2004 ^a^ 2014^e^ | |
| MD | 2004 ^a^ | |
| GUA | 2006 ^c^ 2008 ^c^ 2010 ^c^ 2012 ^c^ 2014 ^c^ | |
| El S | 2006 ^c^ 2008 ^c^ 2010 ^c^ 2012 ^c^ 2014 ^c^ | |
| HON | 2004^c^ 2006 ^c^ 2012 ^c^ 2014 ^c^ | |
| NIC | 2006 ^c^ 2012 ^c^ 2014 ^c^ | |
| CRI | 2006 ^c^ 2008 ^c^ 2010 ^c^ | |
| PAN | 2004 ^c^ 2006 ^c^ | |
| COL | 2012 ^c^ 2014 ^c^ | |
| ECU | 2004 ^c^ 2006 ^c^ 2008 ^c^ 2010 ^c^ 2012 ^c^ 2014 ^c^ | |
| BOL | 2006 ^c^ 2008 ^c^ 2010 ^c^ 2012 ^c^ 2014 ^c^ | |
| PER | 2004 ^a^ 2006 ^c^ 2008 ^c^ 2010 ^a^ 2012 ^c^ 2014 ^c^ | |
| CHI | 2004 ^a e^ | |
| URU | 2004 ^e^ 2006 ^c^ | |
| BRA | 2004 ^a e^ 2008 ^c^ | |
| DOM | 2004 ^c^ 2006 ^c^ 2008 ^c^ 2010 ^c^ 2012 ^c^ 2014 ^c^ | |
| MN | 2006 ^d^ 2010 ^d^ 2014 ^d^ | |
| GH | 2004 ^a^ 2010 ^a^ | |
| ML | 2004 ^a^ | |
| TH | 2001^d^ 2004 ^a^ | |
| ID | 2006 ^d^ 2010 ^d^ | |
| BG | 2010 ^b^ 2012 ^b^ | |
| CZ | 2002 ^b^ 2004 ^b e^ | |
| EE | 2008 ^b^ 2010 ^a^ | |
| HR | 2008 ^b^ 2010 ^b^ 2014 ^e^ | |
| HU | 2004 ^a e^ 2008 ^b^ 2010 ^b^ 2012 ^b^ 2014 ^b e^ | |
| LV | 2004 ^e^ 2006 ^b^ 2008 ^b^ | |
| PL | 2002 ^b^ 2004 ^a e^ 2006 ^b^ 2008 ^b^ 2010 ^b^ 2012 ^b^ 2014 ^b e^ | |
| PT | 2002 ^b^ 2004 ^b e^ 2006 ^b^ | |
| RO | 2008 ^b^ 2010 ^a^ | |
| SI | 2002 ^b^ 2004 ^a b e^ | |
| SK | 2004 ^b e^ 2006 ^b^ 2010 ^b^ | |
| TR | 2010 ^a^ 2014 ^e^ | |
| LT | 2008 ^b^ 2010 ^b^ 2012 ^b^ | |
| S-KR | 2001 ^d^ | |
| MX | 2004 ^a c e^ 2010 ^a c^ 2012 ^c^ 2014 ^c^ | |
| CRI | 2012 ^c^ 2014 ^c^ | |
| PAN | 2008 ^c^ 2010 ^c^ 2012 ^c^ 2014 ^c^ | |
| CHI | 2006 ^c^ 2008 ^c^ 2010 ^a c^ 2012 ^c^ 2014 ^c e^ | |
| URU | 2008 ^c^ 2010 ^a c^ 2012 ^c^ 2014 ^c^ | |
| BRA | 2010 ^a c^ 2012 ^c^ 2014 ^c^ | |
| VEN | 2014 ^c e^ | |
| ARG | 2008 ^c^ 2010 ^a c^ 2012 ^c^ 2014 ^c^ | |
| TT | 2004 ^a^ |  |
| AT | 2002 ^b^ 2004 ^b e^ | |
| BE | 2002 ^b^ 2004 ^b e^ 2006 ^b^ | |
| CY | 2004 ^a e^ 2006 ^b^ 2010 ^a b^ 2012 ^b^ | |
| CZ | 2008 ^b^ 2010 ^b^ 2012 ^b^ 2014 ^b e^ | |
| DE | 2002 ^b^ 2004 ^a b e^ 2006 ^b^ | |
| DK | 2002 ^b^ 2004 ^b e^ | |
| EE | 2012 ^b^ | |
| ES | 2002 ^b^ 2004 ^b e^ 2006 ^b^ 2008 ^b^ 2010 ^a b^ 2012 ^b^ 2014 ^b e^ | |
| FI | 2002 ^b^ 2004 ^a b e^ 2006 ^b^ | |
| FR | 2004 ^a b e^ 2006 ^b^ 2008 ^b^ 2010 ^b^ 2012 ^b^ | |
| GB | 2002 ^b^ 2004 ^a b e^ 2010 ^b^ 2012 ^b^ | |
| GR | 2002 ^b^ 2004 ^b^ 2008 ^b^ 2010 ^b^ | |
| IL | 2004 ^e^ 2008 ^b^ 2010 ^b^ 2012 ^b^ 2014 ^e^ | |
| IT | 2002 ^b^ 2004 ^a b^ 2012 ^b^ | |
| PT | 2008 ^b^ 2012 ^b^ 2014 ^b e^ | |
| SE | 2002 ^b^ 2004 ^a b e^ | |
| SI | 2006 ^b^ 2008 ^b^ 2010 ^a b^ 2012 ^b^ 2014 ^b e^ | |
| S-KR | 2012 ^b^ 2014 ^e^ | |
| LT | 2014 ^b e^ | |
| NZ | 2004 ^e^ 2010 ^a^ | |
| JP | 2001 ^d^ 2004 ^a e^ 2006 ^d^ 2010 ^a^ 2014 ^e^ | |
| S-KR | 2004 ^a e^ 2006 ^d^ 2010 ^a d^ 2014 ^d e^ | |
| TW | 2001 ^d^ 2004 ^e^ 2006 ^d^ | |
| AT | 2006 ^b^ 2008 ^b^ 2014 ^b e^ | |
| BE | 2008 ^b^ 2010 ^b^ 2012 ^b^ 2014 ^b e^ | |
| CH | 2002 ^b^ 2004 ^a b e^ 2006 ^b^ 2008 ^b^ 2010 ^b^ 2012 ^b^ 2014 ^b e^ | |
| DE | 2008 ^b^ 2010 ^a b^ 2012 ^b^ 2014 ^b e^ | |
| DK | 2006 ^b^ 2008 ^b^ 2010 ^b^ 2012 ^b^ 2014 ^b e^ | |
| FI | 2008 ^b^ 2010 ^b^ 2012 ^b^ 2014 ^b e^ | |
| FR | 2014 ^b e^ | |
| GB | 2006 ^b^ 2008 ^b^ 2014 ^b e^ | |
| IE | 2004 ^b e^ 2006 ^b^ 2008 ^b^ 2010 ^b^ 2012 ^b^ 2014 ^b^ | |
| IS | 2004 ^b^ 2012 ^b^ 2014 ^e^ | |
| LU | 2002 ^b^ 2004 ^b^ | |
| NL | 2002 ^b^ 2004 ^a b e^ 2006 ^b^ 2008 ^b^ 2010 ^a b^ 2012 ^b^ 2014 ^b e^ | |
| NO | 2002 ^b^ 2004 ^b e^ 2006 ^b^ 2008 ^b^ 2010 ^b^ 2012 ^b^ 2014 ^b e^ | |
| SE | 2006 ^b^ 2008 ^b^ 2010 ^a b^ 2012 ^b^ 2014 ^b e^ | |
| CA | 2004 ^a e^ 2010 ^c^ 2012 ^c^ | |
| US | 2004 ^a e^ 2010 ^a c^ 2012 ^c^ 2014 ^c e^ | |
| AU | 2004 ^a e^ 2010 ^a^ 2014 ^e^ | |
| TW | 2010 ^a d^ 2014 ^d e^ | |

^a^ = World Values Study (WVS); b = European Social Survey (ESS); c = Latin American Public Opinion Project (LAPOP); d = Asian barometer (ASIAN); e = International Social Survey Program (ISSP).
