# Supplementary material for: The Income Gap in Voting: Moderating Effects of Income Inequality and Clientelism
Source: Polit Behav. 2020 Oct 12;44(3):1203–23. doi: 10.1007/s11109-020-09652-z (PMC9433354; doi:10.1007/s11109-020-09652-z)
Supplement: Supplementary file 2 — Supplementary file2 (DOCX 32 kb) [file 11109_2020_9652_MOESM2_ESM.docx]

# Appendix 2. Overview of harmonization procedures for individual-level variables

## Relative income

| **Survey(year)** | **Original measure** | | | **Harmonized relative income** |
| --- | --- | --- | --- | --- |
| **ESS** |  | | |  |
| **2002-2006** | *Using this card, if you add up the income from all sources, which letter describes your household’s total net income? If you don’t know the exact figure, please give an estimate. Use the part of the card that you know best: weekly, monthly or annual income.* (note: below the value labels are shown for monthly household income) | | | |
|  | J. Less than €150 | | | * |
|  | R. €150 to under €300 | | | * |
|  | C. €300 to under €500 | | | * |
|  | M. €500 to under €1000 | | | * |
|  | F. €1000 to under €1500 | | | * |
|  | S. €1500 to under €2000 | | | * |
|  | K. €2000 to under €2500 | | | * |
|  | P. €2500 to under €3000 | | | * |
|  | D. €3000 to under €5000 | | | * |
|  | H. €5000 to under €7500 | | | * |
|  | U. €7500 to under €10000 | | | * |
|  | N. €1000 or more | | | * |
|  | *Note: Recoding the income categories into income quintiles required the following steps.  Step 1: A new variable was created on which respondents score the mean value of the income category they chose. For example, if a respondent chose category R (€150-€300), this respondent scores 225 on the new income variable.  Step 2: The relative income variable was created by dividing the scores on this new income variable into *within-country* income quintiles. These quintiles are thus based on the survey sample and not based on the actual income distribution in the country. This implies that the translation from the raw categories (as presented above) into income quintiles differs between countries. For example, respondents from Norway who chose categories J, R, C, M, F, S or K were all assigned to the lowest income quintile in their country, whereas from Slovakia only those respondents who chose categories J or R were assigned to the lowest income quintile in their country. These quintiles are based on the survey sample and not based on the actual income distribution in the country. | | | |
| **2002-2006** | *Using this card, please tell me which letter describes your household’s total income, after tax and compulsory deductions, from all sources? If you don’t know the exact figure, please give an estimate. Use the part of the card that you know best: weekly, monthly or annual income.* | | | |
|  | J.1^st^ decile | | 1. Lowest quintile | |
|  | R. 2^nd^ decile | | 1. Lowest quintile | |
|  | C. 3^rd^ decile | | 2. 2^nd^ quintile | |
|  | M. 4^th^ decile | | 2. 2^nd^ quintile | |
|  | F. 5^th^ decile | | 3. 3^rd^ quintile | |
|  | S. 6^th^ decile | | 3. 3^rd^ quintile | |
|  | K. 7^th^ decile | | 4. 4^th^ quintile | |
|  | P. 8^th^ decile | | 4. 4^th^ quintile | |
|  | D. 9^th^ decile | | 5. 5^th^ quintile | |
|  | H. 10^th^ decile | | 5. 5^th^ quintile | |
|  | Note: these categories on the interview cards in ESS round 4-8 were national categories based on deciles of the actual household income range in the given country. Therefore they were country-specific, and the scores were harmonized into income deciles in the ESS dataset. | | | |
|  |  | | |  |
| **LAPOP** |  | | | |
| **2002-2012** | *Into which of the following income ranges does the total monthly income of this household fit, including remittances from abroad and the income of all the working adults and children?* | | | |
|  | 0. No income | 1. Lowest quintile | | |
|  | 1. First decile | 1. Lowest quintile | | |
|  | 2. Second decile | 1. Lowest quintile | | |
|  | 3. Third decile | 2. 2^nd^ quintile | | |
|  | 4. Fourth decile | 2. 2^nd^ quintile | | |
|  | 5. Fifth decile | 3. 3^rd^ quintile | | |
|  | 6. Sixth decile | 3. 3^rd^ quintile | | |
|  | 7. Seventh decile | 4. 4^th^ quintile | | |
|  | 8. Eighth decile | 4. 4^th^ quintile | | |
|  | 9. Ninth decile | 5. Highest quintile | | |
|  | 10. Tenth decile | 5. Highest quintile | | |
|  | Note: The exact income categories that were used in the LAPOP questionnaires are based on the currency and distribution of the specific country, and thus they differ between countries. | | | |
| **2014-2016** | *Into which of the following income ranges does the total monthly income of this household fit, including remittances from abroad and the income of all the working adults and children?* | | | |
|  | Note: 17 income categories were used to measure respondents’ household income in all countries. The exact categories depend on the currency and income distribution of the specific country, and thus they differ between countries. The relative income variable was created by dividing the scores on the income variable into *within-country* income quintiles, similar to the approach for ESS2002-2006. These quintiles are thus based on the survey sample and not based on the actual income distribution in the country. | | | |
|  |  | | |  |
| **ASIAN** | *Here is a scale of household monthly incomes. We would like to know in what group your household on average is, counting all wages, salaries, pensions, dividends and other incomes that come in before taxes and other deduction. Just give the letter of the group your household income falls into.* | | | |
|  | 1. Lowest quintile | | | 1.Lowest quintile |
|  | 2. 2^nd^ quintile | | | 2. 2^nd^ quintile |
|  | 3. 3^rd^ quintile | | | 3. 3^rd^ quintile |
|  | 4. 4^th^ quintile | | | 4. 4^th^ quintile |
|  | 5. 5^th^ quintile | | | 5.Highest quintile |
|  | Note: Quintiles based on official household income statistics were used to measure income, so the exact categories differ between countries and years based on currencies and income distribution. | | | |
|  |  | | |  |
| **ISSP** | Note: Exact wording of the question differed per country, and country-specific answer categories were used to measure income in ISSP. Therefore the relative income variable was created by dividing the scores on the income variable into *within-country* income quintiles to harmonize these incomes, similar to the approach that was used for ESS2002-2006 and LAPOP 2014-2016. These quintiles are thus based on the survey sample and not based on the actual income distribution in the country. | | | |
|  |  | | | |
| **WVS** | *On this card is a scale of incomes on which 1 indicates the “lowest income decile” and 10 the “highest income decile” in your country. We would like to know in what group your household is. Please, specify the appropriate number, counting all wages, salaries, pensions and other incomes that come in.* | | | |
|  | 1. First decile | 1. Lowest quintile | | |
|  | 2. Second decile | 1. Lowest quintile | | |
|  | 3. Third decile | 2. 2^nd^ quintile | | |
|  | 4. Fourth decile | 2. 2^nd^ quintile | | |
|  | 5. Fifth decile | 3. 3^rd^ quintile | | |
|  | 6. Sixth decile | 3. 3^rd^ quintile | | |
|  | 7. Seventh decile | 4. 4^th^ quintile | | |
|  | 8. Eighth decile | 4. 4^th^ quintile | | |
|  | 9. Ninth decile | 5. Highest quintile | | |
|  | 10. Tenth decile | 5. Highest quintile | | |

## Voting

| **Survey(year)** | **Original variable** | **Harmonized voting** |
| --- | --- | --- |
| **ESS** | *Voted last national election* |  |
|  | 1. Yes | 1. Yes |
|  | 2. No | 0. No |
|  | 3. Not eligible to vote | 0. No |
|  |  |  |
| **WVS**  **2004** | *Did you vote in your country’s most recent elections to the national parliament?* | |
|  | 1. Yes | 1. Yes |
|  | 2. No | 0. No |
|  |  |  |
| **LAPOP** | Voted in last presidential election |  |
|  | 1. No | 0. No |
|  | 2. Yes | 1. Yes |
|  |  |  |
| **ASIAN** |  |  |
| **2001** | *Have you participated in last election?* |  |
|  | 1. No | 0. No |
|  | 2. Yes | 1. Yes |
| **2006** | *Have you voted in the last election?* |  |
|  | 0. Not applicable | missing |
|  | 1. No | 0. No |
|  | 2. Yes | 1. Yes |
|  | 9. Decline to answer | missing |
| **2010** | *Did you vote in the most recent national election?* | |
|  | 0. Not applicable | missing |
|  | 1. No | 0. No |
|  | 2. Yes | 1. Yes |
|  | 3. Not eligible to vote | 0. No |
|  | 8. Can’t choose | missing |
|  | 9. Decline to answer | missing |
| **2014** | *Did you vote in the most recent national election?* | |
|  | 0. Not applicable/not yet eligible to vote | missing |
|  | 1. No | 0. No |
|  | 2. Yes | 1. Yes |
|  | 8. Can’t choose | missing |
|  | 9. Decline to answer | missing |
|  |  |  |
| **ISSP** | *Did you vote in the most recent national election?* | |
|  | 1. Yes | 1. Yes |
|  | 2. No | 0. No |

## Education level

| **Survey(year)** | **Original variable** | **Harmonized education** |
| --- | --- | --- |
| **ESS** |  |  |
| **2002-2008** | *Highest level of education completed* |  |
|  | 1. Less than lower secondary education (ISCED 0-1) | 1. No education – primary education |
|  | 2. Lower secondary education completed (ISCED 2) | 2. Some or lower secondary education |
|  | 3. Upper secondary education completed (ISCED 3) | 3. Completed or higher secondary education |
|  | 4. Post-secondary non-tertiary education completed (ISCED 4) | 4.Some (non-university) tertiary education |
|  | 5. Tertiary education completed (ISCED 5-6) | 5. University |
|  |  |  |
| **2010-2016** | *Highest level of education completed* |  |
|  | 0 Not completed ISCED level 1 | 1. No education – primary education |
|  | 113 ISCED 1, completed primary education | 1. No education – primary education |
|  | 129 Vocational ISCED 2C < 2 years, no access ISCED 3 | 2. Some or lower secondary education |
|  | 212 General/pre-vocational ISCED 2A/2B, access ISCED 3 vocational | 2. Some or lower secondary education |
|  | 213 General ISCED 2A, access ISCED 3A general/all 3 | 2. Some or lower secondary education |
|  | 222 Vocational ISCED 2A/2B, access ISCED 3 vocational | 2. Some or lower secondary education |
|  | 229 Vocational ISCED 3C < 2 years, no access ISCED 5 | 2. Some or lower secondary education |
|  | 311 General ISCED 3 >=2 years, no access ISCED 5 | 3. Completed or higher secondary education |
|  | 312 General ISCED 3A/3B, access ISCED 5B/lower tier 5A | 3. Completed or higher secondary education |
|  | 313 General ISCED 3A, access upper tier ISCED 5A/all 5 | 3. Completed or higher secondary education |
|  | 321 Vocational ISCED 3C >= 2 years, no access ISCED 5 | 3. Completed or higher secondary education |
|  | 322 Vocational ISCED 3A, access ISCED 5B/ lower tier 5A | 3. Completed or higher secondary education |
|  | 323 Vocational ISCED 3A, access upper tier ISCED 5A/all 5 | 3. Completed or higher secondary education |
|  | 412 General ISCED 4A/4B, access ISCED 5B/lower tier 5A | 4.Some (non-university) tertiary education |
|  | 413 General ISCED 4A, access upper tier ISCED 5A/all 5 | 4.Some (non-university) tertiary education |
|  | 421 ISCED 4 programmes without access ISCED 5 | 4.Some (non-university) tertiary education |
|  | 422 Vocational ISCED 4A/4B, access ISCED 5B/lower tier 5A | 4.Some (non-university) tertiary education |
|  | 423 Vocational ISCED 4A, access upper tier ISCED 5A/all 5 | 4.Some (non-university) tertiary education |
|  | 510 ISCED 5A short, intermediate/academic/general tertiary below bachelor | 5. University |
|  | 520 ISCED 5B short, advanced vocational qualifications | 5. University |
|  | 610 ISCED 5A medium, bachelor/equivalent from lower tier tertiary | 5. University |
|  | 620 ISCED 5A medium, bachelor/equivalent from upper/single tier tertiary | 5. University |
|  | 710 ISCED 5A long, master/equivalent from lower tier tertiary | 5. University |
|  | 720 ISCED 5A long, master/equivalent from upper/single tier tertiary | 5. University |
|  | 800 ISCED 6, doctoral degree | 5. University |
|  |  |  |
| **WVS** | *Highest level of education completed* |  |
| **2004** | 1 No formal education | 1. No education – primary education |
|  | 2 Incomplete primary school | 1. No education – primary education |
|  | 3 Complete primary school | 1. No education – primary education |
|  | 4 Incomplete secondary school: technical/vocational type | 2. Some or lower secondary education |
|  | 5 Complete secondary school: technical/vocational type | 3. Completed or higher secondary education |
|  | 6 Incomplete secondary: university-preparatory type | 2. Some or lower secondary education |
|  | 7 Complete secondary: university-preparatory type | 3. Completed or higher secondary education |
|  | 8 Some university-level education, without degree | 4.Some (non-university) tertiary education |
|  | 9 University-level education, with degree | 5. University |
|  |  |  |
| **LAPOP** | *Years of education* |  |
|  | 0 | 1. No education – primary education |
|  | 1-3 | 1. No education – primary education |
|  | 4-6 | 1. No education – primary education |
|  | 7-9 | 2. Some or lower secondary education |
|  | 10-12 | 3. Completed or higher secondary education |
|  | 13-15 | 4.Some (non-university) tertiary education |
|  | 15+ | 5. University |
|  |  |  |
| **ASIAN** | *Highest level of education accomplished* |  |
|  | 1. No formal education | 1. No education – primary education |
|  | 2. Incomplete elementary school | 1. No education – primary education |
|  | 3. Complete elementary school | 1. No education – primary education |
|  | 4. Incomplete secondary school | 2. Some or lower secondary education |
|  | 5. Complete secondary school | 3. Completed or higher secondary education |
|  | 6. Incomplete high school | 2. Some or lower secondary education |
|  | 7. Complete high school | 3. Completed or higher secondary education |
|  | 8. Some university, college education | 4.Some (non-university) tertiary education |
|  | 9. University, college degree | 5. University |
|  | 10. Post graduate degree | 5. University |
|  | |  |
| **ISSP** | *Highest level of education accomplished* |  |
|  | 0. No formal education | 1. No education – primary education |
|  | 1. Primary school | 1. No education – primary education |
|  | 2. Lower secondary | 2. Some or lower secondary education |
|  | 3. Upper secondary | 3. Completed or higher secondary education |
|  | 4. Post-secondary, non-tertiary | 4.Some (non-university) tertiary education |
|  | 5. Lower level tertiary | 4.Some (non-university) tertiary education |
|  | 6. Upper level tertiary | 5. University |
