# Supplementary material for: The Income Gap in Voting: Moderating Effects of Income Inequality and Clientelism
Source: Polit Behav. 2020 Oct 12;44(3):1203–23. doi: 10.1007/s11109-020-09652-z (PMC9433354; doi:10.1007/s11109-020-09652-z)

**Appendix 3. Comparisons of the effects of relative income on voting between surveys within country-years**
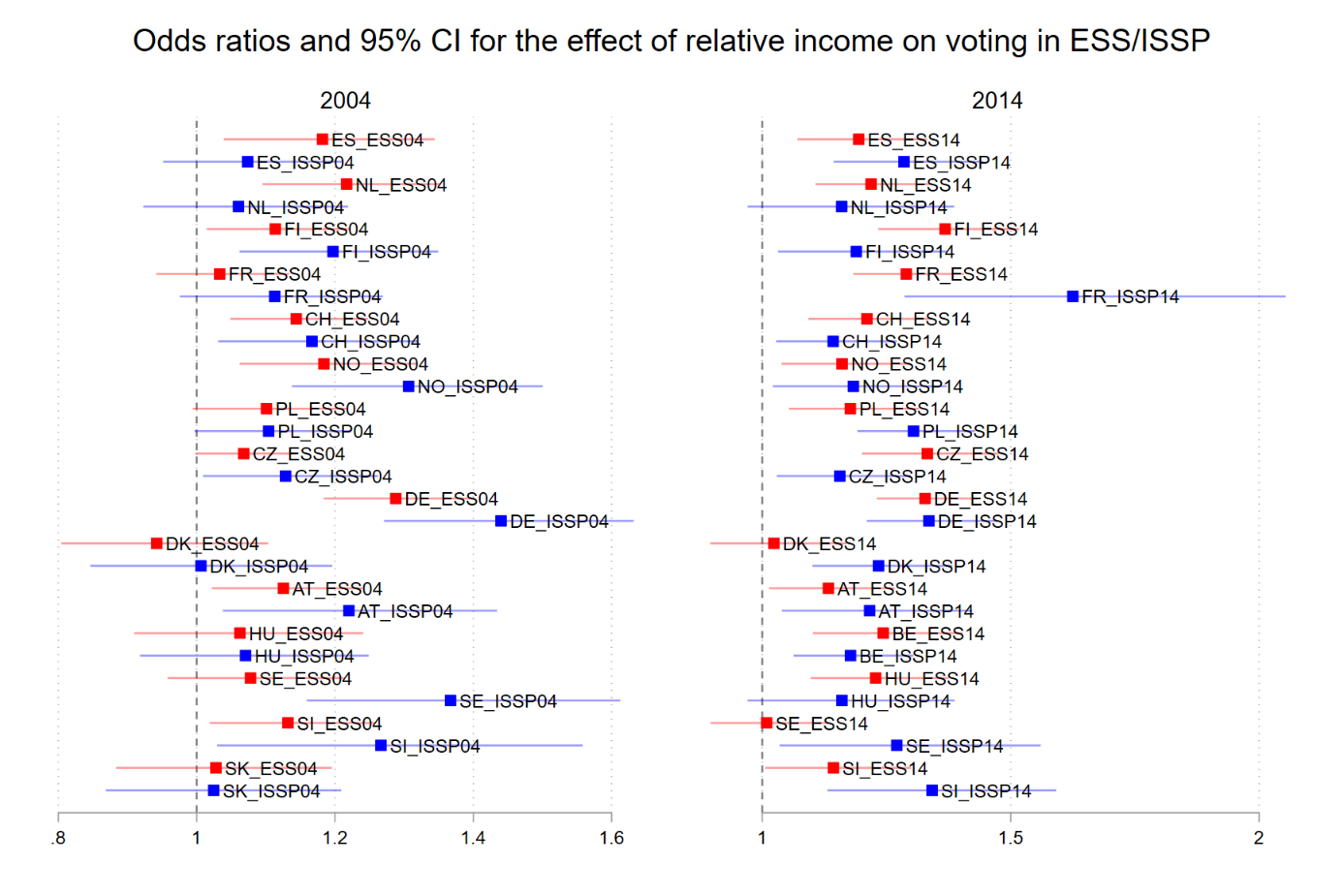


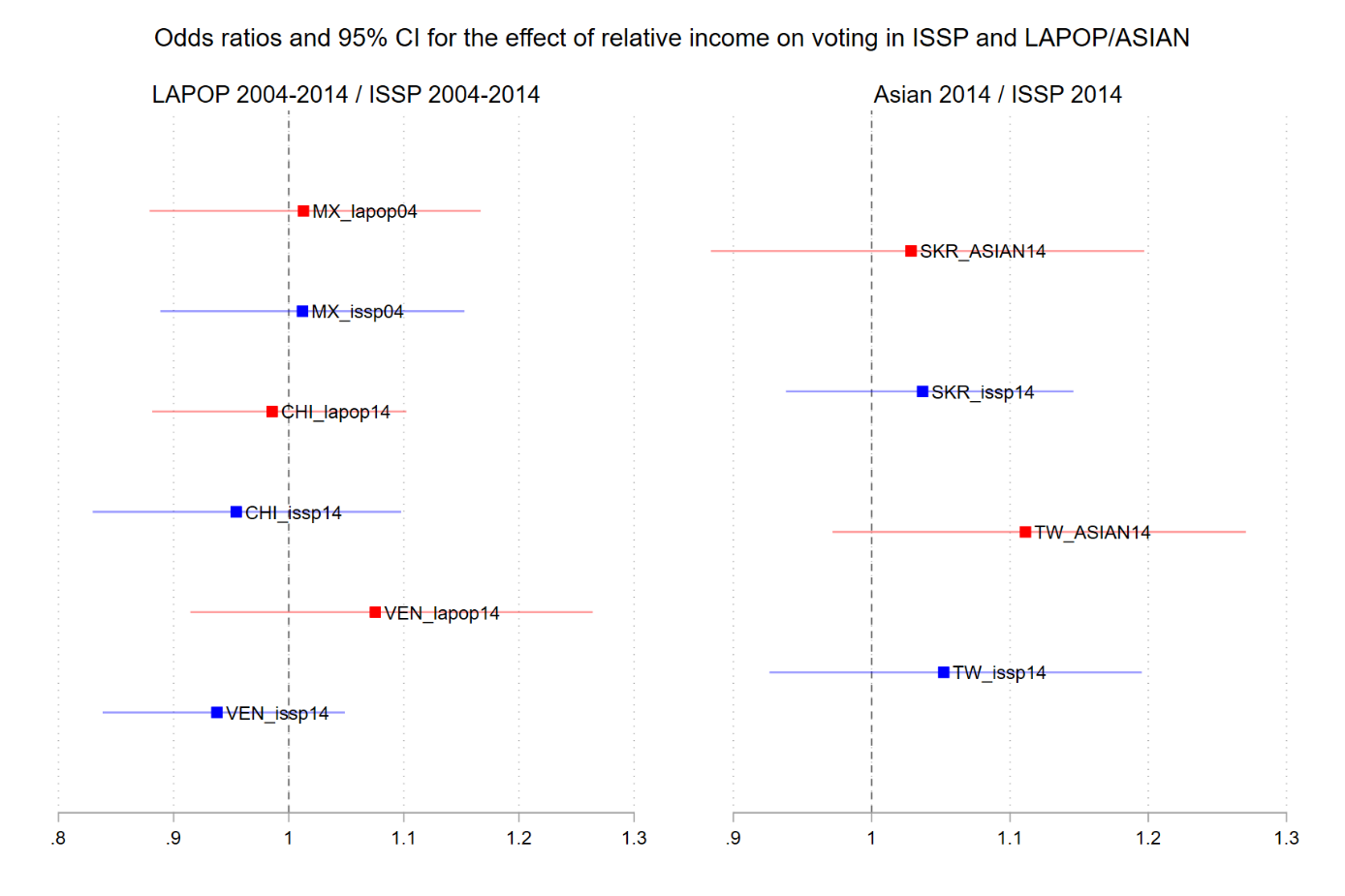


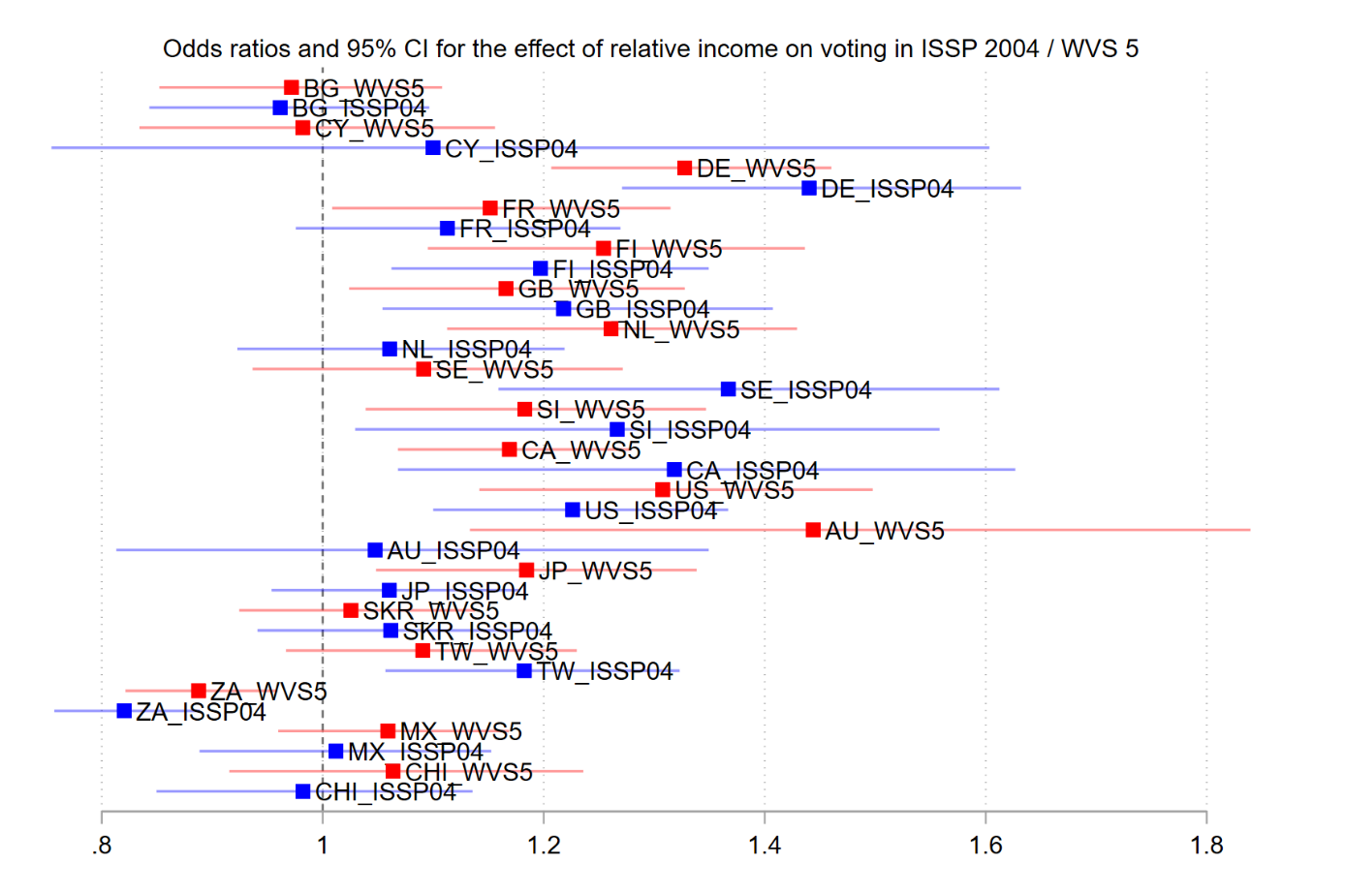


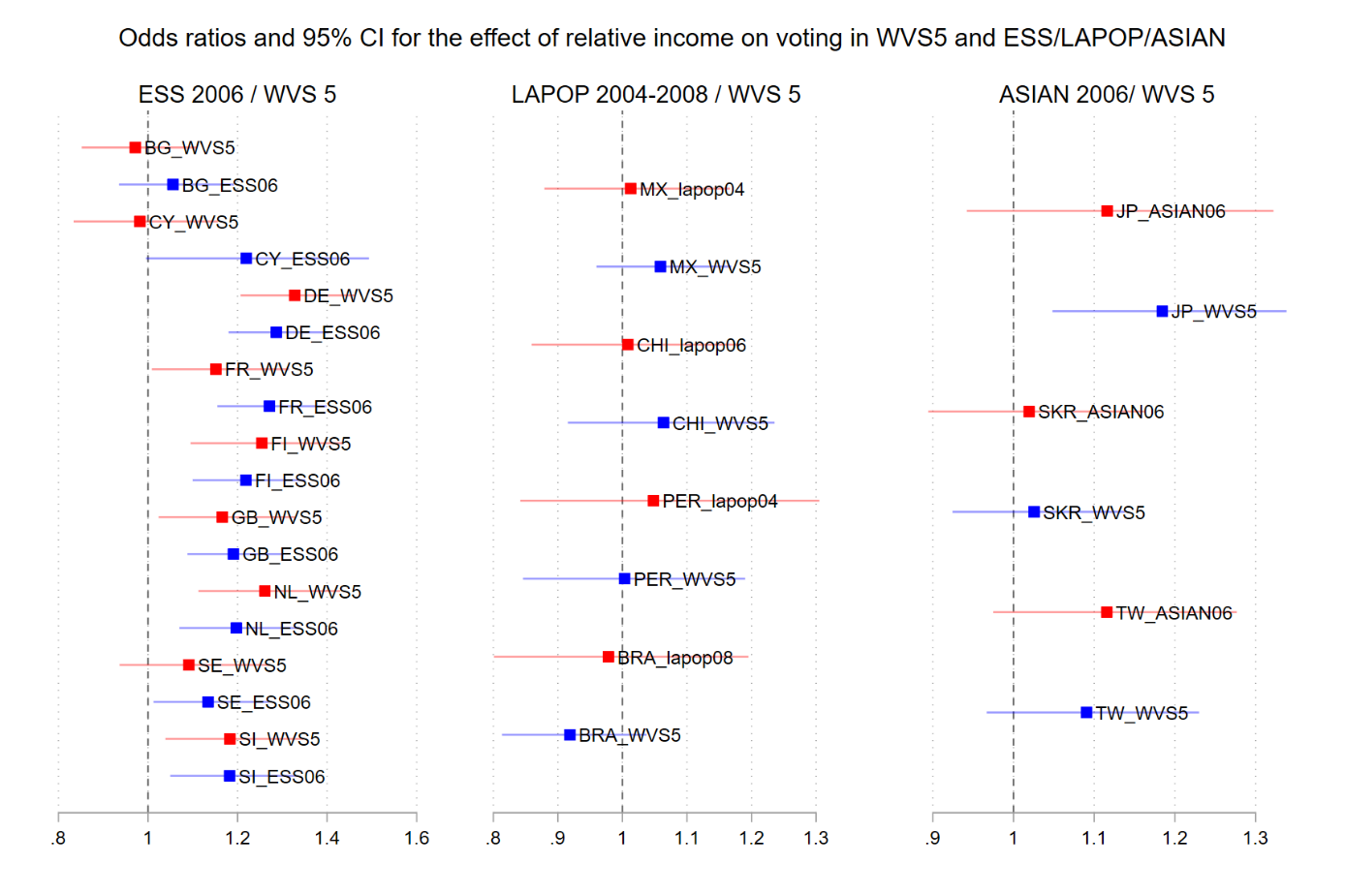

Supplement: Supplementary file 3 — Supplementary file3 (DOCX 1198 kb) [file 11109_2020_9652_MOESM3_ESM.docx]
